# Being a Young Carer in Portugal: The Impact of Caring on Adolescents’ Life Satisfaction

**DOI:** 10.3390/ijerph20217017

**Published:** 2023-11-03

**Authors:** Ana Meireles, Sofia Marques, Sara Faria, Joana Correia Lopes, Ana Ribas Teixeira, Bruno Alves, Saul Becker

**Affiliations:** 1 Centro de Investigação em Psicologia para o Desenvolvimento, Universidade Lusíada, 4100-348 Porto, Portugal; smarques@por.ulusiada.pt (S.M.); sarafaria22@gmail.com (S.F.); psi.joanacorreialopes@gmail.com (J.C.L.); 2 Portincarers Associação Cuidadores Portugal, 4200-249 Porto, Portugal; ana.ribasteixeira@gmail.com (A.R.T.); pcjbta@gmail.com (B.A.); 3 Faculty of Health and Education, Manchester Metropolitan University, Manchester M15 6BX, UK; s.becker@mmu.ac.uk

**Keywords:** young carers, informal carers, life satisfaction, social support, family functioning, academic functioning, caring activities

## Abstract

Caring for an ill or disabled relative can present significant challenges that may exceed the personal resources of the caregiver. Young carers (YCs) often take on this role, providing support to family members or friends, which can have far-reaching effects on various aspects of their lives. This study involved 235 adolescents, 106 YCs, and 129 non-carers (NCs), who completed questionnaires assessing life satisfaction, satisfaction with social support, family functioning, academic functioning, and caregiving activities. Tests of group differences (MANOVA and MANCOVA controlling for age) showed YCs had more caregiving activities than NCs (as expected) and, critically, significantly lower life satisfaction. Hierarchical regressions with the YCS subsample showed academic functioning, social support, and the negative impact of caregiving were associated with life satisfaction, and that the negative influence of caregiving was linked to family functioning and the quantity of caregiving activities. For NCs, academic functioning, satisfaction with social support, and family functioning were associated with life satisfaction. In conclusion, caregiving in adolescents appears to be linked to lower life satisfaction, but this effect is determined by their social support, academic functioning, and negative impact of caring, which in turn depends on their family functioning and amount of caring activities.

## 1. Introduction

Caring for individuals with disabilities presents a range of challenges that can exceed the carer’s personal resources [1], leading to potentially significant negative impacts (physical, psychological, social, and economic) on the lives of the carer and the person being cared for. Young carers (YCs) are defined as young people who provide informal (unpaid) care, assistance, or support to family members or friends. These family members are usually parents or grandparents, but they can also be siblings or other relatives [2]. YCs often take on substantial caregiving responsibilities, assuming roles that would typically be associated with adults [3]. Although the age range of YCs varies in the literature—a YC can be as young as 5 years old up to the age of 26 [4,5]—a typical distinction is between carers under 18 and young adult carers up to age 25 [6]. However, research to date has mostly focused on adolescent YCs [4]. While around 6–8% of adolescents may be classified as YCs [7], this group remains largely hidden [8] with their specific needs and challenges often overlooked in research and policy in most countries [6]. The prevalence of YCs in Portugal may be higher than that observed in different European countries and abroad [9,10,11,12,13,14] due to the aging of the population [15], mental health [16] and disability issues [15], along with cultural aspects of family role and limitations on formal support. In addition, high rates of single-parent and extended families [15] also put young people at the first level of care for the disabled. However, Portuguese YCs existence and needs remain virtually unknown and neglected, showing a low level of awareness and policy response, according to Leu and Becker’s [17] country classification and criteria, as no legal reference or national support exists for them. In Portugal, Adults Informal Carers were only legally recognized in 2019, and there is no reference or data on YCs. Though awareness of their situation by professionals, families, and YCs themselves remains low overall, resulting in a largely hidden group [8].

### 1.1. Negative Consequences of Being a Young Carer

Reports of worse physical and mental health are recurring themes in the YCs literature. The impact of caregiving on their physical health can be demonstrated by YCs complaints of tiredness, exhaustion, poor sleep, headaches, back pain, physical exertion, injuries, suffering from their illnesses, and impacts on diet and exercise [3,4,12,18,19].

Caring responsibilities can have profound implications for the mental health of YCs [3,4,20,21]. A systematic review conducted by Fleitas Alfonzo, Singh, Disney, Ervin and King [21] indeed confirmed a consistent association between being a YC and experiencing poor mental health, including depression, anxiety, and other emotional problems. The challenges that YCs face can encompass feelings of worry, anger, and resentment towards the relative they care for [19,22,23,24,25,26], as well as emotional experiences such as sadness and disappointment [27]. Additionally, YCs often grapple with feelings of guilt for not doing enough to help [22] and can find themselves overwhelmed and exhausted [3,22,24]. The impact on mental wellbeing can extend to difficulties with sleeping and eating, self-harm, substance use, and suicidal ideation [22]. YCs may also be susceptible to self-mutilation, peer rejection, and bullying [3,19,22,28,29,30]. These challenges are consistently associated with lower levels of self-esteem, life satisfaction, happiness, and overall wellbeing [22,30,31,32,33], as well as lower quality of life [34] when compared to NCs. Disturbingly, research indicates these consequences can manifest as young as 10 to 11 years old [30], with YCs showing poorer health and less happiness in their lives than their non-carer peers. Furthermore, these impacts on mental health appear not to be limited to the period of caregiving but can persist even four years later, as revealed by a longitudinal study by King et al. [35].

Additionally, caring responsibilities can significantly hinder academic engagement and performance in YCs [25,26,36]. This can arise from difficulties concentrating in class, struggling to meet deadlines, having limited time to complete homework, and school absenteeism due to caregiving obligations [37], as well as poorer educational aspirations and outcomes [30]. The combination of these two roles (caring and learning) may create an overwhelming burden for these young people [38], which may be reflected in feelings of lack of control over their life expectations, leading to conflicts, particularly when faced with contradictory or incompatible needs [27].

### 1.2. Positive Consequences of Being a Young Carer

Despite the challenges they face, it is important to acknowledge that not all YCs experience difficulties in health, wellbeing, or education. For some, the experience of caring is beneficial, leading to personal growth, maturity, and competence [39]. Research highlights the existence of positive consequences associated with being a caregiver [24,25,40]. These positive outcomes include the development of advanced coping mechanisms, maturity, and a sense of responsibility [3,19,41,42,43]. Caring can also help YCs acquire a range of valuable skills and values, including compassion and empathy [3,24,44,45], the ability to manage challenging situations and resilience [41,44,46,47], independence and autonomy [25], knowledge and skills about health and advocacy [25], social skills, interpersonal relationships, proximity to the person cared for [3], self-efficacy, self-confidence, tolerance, and self-esteem [25], setting priorities, and seeking out services of family and community support.

Several studies have demonstrated that YCs can derive value from their caregiving role and ability to support loved ones. Such studies have shown YCs often report feelings of satisfaction, pride, protection, and gratification [48], along with a sense of fulfilment [22] or accomplishment [49] and a strong sense of purpose [38]. Additionally, caregiving can foster closer relationships between the YCs and the person(s) for whom they care. These positive aspects can moderate the relationship between ‘caregiver burden’ and YCs mental health and wellbeing [22]. Similarly, Gough and Gulliford [49] concluded that YCs are often particularly resilient and use a benefit-seeking orientation to form a sense of satisfaction and accomplishment from their roles, along with having practical coping strategies and a strong sense of agency and connectedness that also promoted better outcomes for YCs. Benefit-finding was shown to directly enhance better mental wellbeing and indirectly influence wellbeing by promoting better coping and reducing feelings of helplessness [50].

### 1.3. Influencing Factors

Perhaps unsurprisingly, the relationship between the amount of caring activity and measures of wellbeing and mental health appears to be complex. Rather than assuming a straightforward linear relationship, this relationship is likely to be influenced by various factors. Joseph, Sempik, Leu and Becker [39] acknowledge that research on this topic has tended to overlook moderating factors and lack theoretical sophistication. It is essential to recognize that caring occurs within a cultural context and is shaped by diverse support systems across schools, communities, and helping professions [51]. Therefore, how caring activity relates to health and wellbeing is expected to be dependent on multiple and interrelated factors.

#### 1.3.1. Socio-Demographic Factors

Research shows that being a YC and its impacts depend on a range of factors. These factors include age and gender [20,22,52,53], living arrangements and financial status [22,52,54], ethnicity [53,55], the identity of the ill/disabled relative being supported [20,22,33], the nature of the illness or disability [20,22,26], the level of dependency and the need for assistance [22,26], and family structure and support [26]. Being a YC can become potentially ‘damaging’ when it is persistent over the long term and excessive, with responsibilities that are disproportionate to the capabilities of a child’s age or level of maturity. This may involve caring for extended durations and starting at a younger age [24,53,56]. Girls and older YCs also tend to have higher caring demands and responsibilities [22] and are more likely to report issues with their mental health [22,52,53,57]. Age also influences the degree to which YCs are aware of their role in the family structure, with adolescents being more aware than younger children of how their caring responsibilities impact their lives [22].

Research suggests that the negative mental health outcomes of caring are more likely when YCs provide personal care, including more intimate forms of care [22,58], and engage in emotional caring tasks [22] that are perceived as more burdensome compared to instrumental caring tasks related to household chores. In general, there is a dose-response relationship between care and mental health [35,57], with stronger effects seen among those who provide daily and more intensive care [35]. Bou [22] also found that YCs burden was directly correlated with stress and inversely related to their quality of life and life satisfaction, and Pakenham and Cox [59] emphasize that higher caregiving responsibilities have both direct and indirect adverse effects on YCs mental health. Many YCs only realized that their own needs had been neglected after they stopped being carers, which ultimately prevented them from taking the opportunity to ask for help, to practice self-care, and to practice self-compassion [12]. In fact, various limitations to YCs self-care have been observed [47].

#### 1.3.2. Social Support

Social support and the quality of relationships play an important role in moderating the health and wellbeing of carers [27,60,61]. The availability of social resources has been found to be highly predictive of YCs adjustment and positive outcomes [3,42]. For example, Pakenham, Chiu, Bursnall and Cannon [40] found that social support increased YCs life satisfaction and, indeed, was the strongest and most consistent predictor of social adjustment. However, YCs might reduce their involvement in social activities, either because of the stigma associated with caregiving or the added burden in the form of perceived stress [62]. This reduction may lead them to allocate less time for personal development, socializing with friends, and other social and leisure activities [26]. As a result, social isolation, social stigma, and bullying experiences may intensify [22,26]. YCs often report feeling ‘different’ and misunderstood by their peers, leading them to withdraw from their circle of friends and conceal their identity as caregiver [37]. Self-stigma can hinder the formation of peer relationships, as YCs may be hesitant to share their circumstances due to privacy concerns or the fear of illness-related stigma [22]. Some YCs may even hide their relatives’ conditions from others to avoid being identified as ‘young carers’ [63]. Thus, for some YCs, caring can hinder the development of friendships by requiring sacrifices in social opportunities, making them feel “older than their age”, and contributing to loneliness [22].

Guggiari, Fatton, Becker, Lewis, Casu, Hoefman, Hanson, Santini, Boccaletti, Nap, Hlebec, Wirth and Leu [52] found associations between receiving support and visibility from their school and YCs health-related quality of life; moreover, YCs who reported that their school knew about their carer role also reported fewer mental health issues [52] and, when YCs did seek support, they better adjusted to caring [22]. These associations seem to be complex, as the disclosure about their caring role and YCs social support appears to be related, and YCs recognize that identifying as a YC could help them gain recognition for their roles and obtain the support they need [22].

Support from schools and teachers plays an important role in YCs wellbeing and development, and while many YCs seemed to enjoy going to school as it provides them with a break from caring and the possibility to socialize [36], some of them face bullying and harassment [22]. YCs relationships with teachers varied too, with some feeling supported and accommodated and others perceiving teachers as insensitive to their caring situations and their impact on their studies or misunderstanding their difficulties [19]. In general, evidence suggests that even if not all YCs inform their school and teachers of their caring role [38], when YCs did confide in teachers, their academic performance and enjoyment of education improved [22]. Though wellbeing, social support, and satisfaction of adolescents’ basic psychological needs at school (i.e., autonomy, competence, and relatedness) do not have a linear relationship [64].

To understand the experiences of YCs, it is essential to consider the role of family variables. In general, the way families function can provide an important source of emotional and material support for their members [65]. Family functioning encompasses how family members communicate, relate to one another, make decisions, and resolve problems [66]. Adaptive family functioning has been shown to contribute to positive development in young people, particularly in terms of their mental health, self-esteem, and life satisfaction. Conversely, problems with family functioning can negatively influence the adjustment of young people and have been associated with symptoms of depression and anxiety [67]. The family unit is, therefore, a unique context for learning how to interact, adapt, and communicate, and where positive or negative emotions give meaning to the feeling of being and belonging [68]. While there are several sources of stress that can contribute to the burden felt by families (for example, illness or disability) [69], families often employ effective strategies to address stressful events and circumstances. These strategies may include the use of various resources, both internal, such as open communication and mutual support, and external, such as seeking social support [70]. Surprisingly, the functioning of YCs families has not been a primary focus in research [22] or support for YCs ([45], for an exception). However, when YCs feel supported and acknowledged by their family members, including the person they care for, they report that caring actually improves their family closeness and their relationship with the person(s) for whom they provide care. In contrast, when they lacked this support and recognition, they tended to view their caregiving experience negatively [22]. Moreover, recognition for their caregiving role and support from relatives and the person they cared for predicted better wellbeing [27].

### 1.4. The Present Study

It is essential to recognize that caregiving can present both challenges and opportunities for positive development. To empower YCs and help them overcome their vulnerabilities, it is necessary to better understand the psychological factors that influence their caregiving experiences. By considering key stressors related to caregiving, such as the extent of caregiving responsibilities, and also taking into account contextual factors like academic and family functioning, it is possible to gain insights into how caregiving impacts the wellbeing and mental health of YCs [51]. Examining the subjective experiences of caregiving, which go beyond merely assessing intensity or quantity, is essential for understanding its effects on children and young people. By doing so, it is possible to better understand how these factors shape the relationship between the caring role and other relevant life outcomes, which may not be as previously assumed in some past studies [39].

Recognizing the prevailing focus on measuring negative mental health outcomes, such as depression, anxiety, and other mental or emotional problems among YCs, the current study sought to consider positive indicators and mediating factors. Specifically, this study had two objectives: (1) to assess how YCs compare to their peers without caring roles in terms of life satisfaction, and (2) to develop a model that helps to explain factors contributing to life satisfaction for YCs (compared to NCs), taking into account the social support received, their family and academic functioning, the amount of caring activities they perform, and, for caregivers only, the subjective impact of being a caregiver. Additionally, we also aim to (3) understand predictors of the negative impact of caring on YCs.

## 2. Materials and Methods

### 2.1. Recruitment and Data Collection

#### 2.1.1. Young Carers Group

Data for YCs were collected between April and June 2023 as a part of “Projecto Jovens Cuidadores” (POISE 39-4639-FSE-000681). The “Projecto Jovens Cuidadores” was led by Portincarers—Associação Cuidadores de Portugal, the first national association of carers to work with and for YCs in Portugal. The project was funded by Portugal Inovação Social—partnerships for impact—and by Vila Nova de Gaia City Council social investors; with the collaboration of the Portuguese Sports and Youth Institute. The project aimed to identify and support YCs in different areas, such as assessing their needs, providing psychological support, and promoting their quality of life, involving YCs, their families, schools, local institutions, and authorities. The project team has a solid academic background, with members ranging from senior researchers to junior beginning researchers in psychology and nursing. Part of this team carried out this study in parallel with their support for YCs across 17 schools in Vila Nova de Gaia, Portugal. This study was approved by the Ethics Committee of the corresponding author’s affiliated university.

After contacting and recruiting schools for this study, members of the research team conducted a total of 419 screening/presentation sessions across the 17 schools. In these sessions, the project’s aims were presented to students, and preliminary data that could help identify YCs were collected. Specifically, students were invited to respond individually to questions on (i) the existence of ill/disabled relative(s) in their family and (ii) the care they provided. Of the 9015 participants who provided this information, 951 students (10.6%) were identified as YCs and consequently invited to participate in this study. Of these individuals, 106 agreed to participate in data collection and provided informed consent from parents or a legal guardian, as determined by Portuguese law. The YC status of these participants was confirmed by interviewing the YCs and their parents (or other legal representatives) either at school, online, or at their homes. Before completing the survey, all participating adolescents were informed about this study aims, confidentiality issues, and the voluntary nature of participation. Finally, YCs were sent an online questionnaire to complete, including all this study’s measures.

#### 2.1.2. Non-Carers Group

To recruit the sample of NCs, we used two schools, selected based on their convenience, from the wider sample of 17. In these two schools, both YCs and NCs were invited to participate in this study. Of the NCs invited, 129 agreed to participate and obtained informed consent from a legal guardian. These students then completed a paper version of the questionnaires in a classroom context, including the same measures as the YCs sample except for the subjective impact of caring, lasting approximately 25 min. Prior to completing the survey, all participating adolescents were informed about this study aims, confidentiality issues, and the voluntary nature of participation.

### 2.2. Measures

#### 2.2.1. Socio-Demographic and Caring Characteristics

Participants completed a socio-demographic questionnaire with items assessing gender, age, school year, household composition and structure, and mother and father education level as an indication of socioeconomic status. Specific information related to the YCs role was also collected: cared person(s), number of cared persons, years of caring role, and number of hours on caring tasks per week.

#### 2.2.2. Life Satisfaction

We assessed adolescents’ satisfaction with life using the Portuguese translation of the Brief Multidimensional Students’ Life Satisfaction Scale (BMSLSS), which has been validated for use in Portuguese samples [71,72]. The six items of this scale evaluate satisfaction with five specific domains relevant for adolescents (family, friends, school, oneself, and home environment) and life ‘in general’. Each item is rated on a 7-point Likert scale from 0 (Terrible) to 6 (Fantastic). As a unidimensional construct, scores across the six items are averaged to compute a composite life satisfaction score, with higher scores indicating higher life satisfaction. The scale shows good internal consistency with a Cronbach’s alpha coefficient of 0.81.

#### 2.2.3. Satisfaction with Social Support

To assess adolescents’ satisfaction with their social support, we used the 12-item Satisfaction with Social Support Scale (SSSS), the original version of which is in Portuguese [73]. SSSS items, each rated using a 5-point Likert scale from 1 (Strongly Disagree) to 5 (Strongly Agree), are organized in two dimensions: Satisfaction with Social Support (six items) (e.g., I am satisfied with the number of friends I have) and Need for Activities connected to Social Support (six items) (e.g., I would like to participate in more organized activities (e.g., sports clubs, scouts)). As an index of total satisfaction with social support, we computed an average score across the 12 items, with higher values representing greater satisfaction. Cronbach’s alpha yielded acceptable consistency results for the total score of the SSSS (α = 0.81).

#### 2.2.4. Family Functioning

We evaluated family functioning using the Portuguese translation of the Systemic Clinical Outcome Routine Evaluation (SCORE-15) [70,74]. The SCORE-15 has 15 items that measure various aspects of family functioning that are sensitive to therapeutic change. These can be grouped according to three dimensions: Family resources (e.g., We are good at finding new ways of dealing with difficulties), Communication in the family (e.g., My family often doesn’t get along tell the truth with each other), and Family Difficulties (e.g., We feel very unhappy in our family). Items are rated from 1 (Describes us very well) to 5 (Describes us very poorly). A total score was computed, with lower scores indicating better family functioning [70,74]. Concerning the psychometric characteristics, there is good internal consistency both for the total scale (α = 0.91), and its dimensions (αs > 0.81).

#### 2.2.5. Academic Functioning

We used a 9-item questionnaire that was developed for this study (research version, [75]) to assess major indicators of adolescents’ academic accomplishment and ability to fulfill academic behavior. Items include a self-report on academic performance (grades), punctuality and assiduity, homework attainment and home studying, willingness to go to school, and ability to concentrate in class. Items are scored on a 5-point Likert-type scale from 0 (Very Insufficient) to 4 (Very Good), following terms regularly used in schools to evaluate adolescent students’ performance. Higher values represent better academic functioning. Cronbach’s alpha yielded acceptable consistency results for the total score (α = 0.85).

#### 2.2.6. Caring Activities

We used the Portuguese translation of the Multidimensional Assessment of Caring Activities (MACA-YC18) [41] to calculate the total amount of caring activities carried out both by YCs and NCs. The MACA-YC18 is a self-report questionnaire consisting of 18 items, each scored on a 3-point Likert-type scale from 0 (Never) to 2 (Often), that are distributed across 6 subscales. Specifically, respondents are asked to indicate how often they have carried out each of the following types of jobs in the last month: Domestic Tasks (e.g., clean your own room), Household Management (e.g., take responsibility for buying food), Personal Care (e.g., help the person you care for to take a bath or shower), Emotional Care (e.g., pay attention to the person you care for to make sure that she is fine), Care for Siblings (e.g., take care of your brothers or sisters alone), and Financial/Practical Management (e.g., work part-time to contribute money at home). Responses across the items were summed to a total score, with higher values reflecting more caring activities. The original version of the MACA-YC18 has good psychometric characteristics with acceptable internal consistency for the different subscales [41]. For the present study, internal consistency for the total scale (α = 0.86) and for subscales was also acceptable, ranging from α = 0.65 (domestic tasks) to α = 0.85 (personal care).

#### 2.2.7. Subjective Impact of Caring

To assess the YCs subjective cognitive and emotional impact of caring, we used the Portuguese translation of the Positive and Negative Outcomes of Caring (PANOC-YC20) [41]. The PANOC-YC20 is a self-report questionnaire composed of 2 subscales (positive and negative), each consisting of 10 items that are scored on a 3-point Likert scale from 0 (Never) to 2 (Often). The positive subscale assesses the extent to which the young person positively experiences the role of caregiver (e.g., I feel good about myself when caring for someone). In turn, the negative subscale assesses the extent to which the young person negatively experiences their role as a caregiver (e.g., When caring, I have to do things that make me upset). The original version of the PANOC-YC18 has good internal consistency, with a Cronbach’s alpha coefficient of 0.91 for the positive subscale and 0.92 for the negative subscale [41]. In our study sample, internal consistency was also good for the positive (α = 0.83) and the negative (α = 0.86) subscales.

### 2.3. Participants

The final analytic sample comprised 235 adolescents (89 boys and 140 girls), aged between 13 and 18 years old (*M* = 15.18, SD = 1.19), with 106 participants (35 boys and 71 girls; *M*_age_ = 15.5, SD = 1.30) identified as YCs and 129 participants (54 boys and 69 girls; *M*_age_ = 14.9, SD = 1.01) identified as NCs. While the sample of YCs is equally distributed for both levels of education (50%), the sample of NCs has a greater number of basic education students (71.3%) than secondary education students (28.7%), considering the Portuguese education system. Further sample characteristics are presented in Table 1.

As shown in Table 2, most YCs cared for their parents (41.5%) or grandparents (34.9%), and most (85.8%) cared for one person. On average, YCs had been carers for 4.6 years (*SD* = 3.5). Most YCs reported spending 7–14 h a week caring (28.3%) or less (24.5%), but many exceeded this (15.1%). Almost a third of YCs were unable to quantify the number of hours of care they gave per week (32.1%). In terms of the amount of caring activity undertaken, as assessed using the Multidimensional Assessment of Caring Activities Checklist [41], most YCs show high (37.7%) or very high (30.2%) levels, with less showing moderate (22.6%) or low (9.4%) levels.

### 2.4. Data Analysis

Statistical analyses were performed using IBM SPSS version 26.0, assuming an alpha level of 0.05. Descriptive statistics were computed for YCs and NCs separately. Given the proportion of missing data (17% of cases with >5% missing values), we used Multiple Imputation (20 imputations) to replace missing values. Variations across the multiple imputations were minimal, so we opted to use the original sample in the subsequent analyses.

First, we performed preliminary multivariate analyses of variance (MANOVAs) to examine gender differences for all variables within this study. As no gender differences were identified, this variable was not controlled for in the subsequent analyses. Conversely, age showed a positive correlation with family functioning and the amount of caring activities performed. Therefore, it was included as a control in the analyses involving these variables.

Next, we used MANOVA to test for statistical differences between YCs and NCs on a weighted linear combination of multiple dependent variables: life satisfaction, satisfaction with social support, and academic functioning. This was followed by a multivariate analysis of covariance (MANCOVA) to test for statistical differences between YCs and NCs on a weighted linear combination of family functioning and amount of caring activities, controlling for age. Effect sizes for these analyses were estimated with a partial eta squared statistic (*η*_p_^2^), considering *η*_p_^2^ < 0.01 as a low effect, 0.01 < *η*_p_^2^ < 0.06 as a medium effect, and 0.06 < *η*_p_^2^ < 0.14 as a large effect [76].

Next, we used a hierarchical regression approach to assess the extent to which the independent variables were associated with life satisfaction in the YCs group. In this model, Step 1 included age, Step 2 added family functioning, academic functioning, and satisfaction with social support, and Step 3 added the amount of caring activities and the negative impact of caring. In a second analysis, the same approach was taken to understand the variables associated with the negative impacts of caring. In this model, Step 1 included age, Step 2 added family functioning, academic functioning, and satisfaction with social support, and Step 3 added the amount of caring activities. Finally, the hierarchical regression used to test the extent to which the independent variables were associated with life satisfaction was repeated for the NCs group.

## 3. Results

### 3.1. Group Differences: Comparing Young Carers and Non-Carers

First, we tested differences between YCs and NCs across life satisfaction, satisfaction with social support, and academic functioning using MANOVA. According to Wilk’s statistic, there was a significant effect of group across the dependent variables, Λ = 0.79, *F*(5, 133) = 6.89, *p* < 0.001, *η_p_*^2^ = 0.21. Separate univariate tests on the outcome variables revealed significant, albeit weak, group differences in life satisfaction. *F* (1, 137) = 4.85, *p* = 0.045, *η_p_*^2^ = 0.03. There were no significant group differences for satisfaction with social support or academic functioning (Table 3).

Next, we tested differences between YCs and NCs across family functioning and the amount of caring activities controlling for age. According to Wilk’s statistic, there was a significant effect of group, Λ = 0.83, *F*(2, 206) = 7.59, *p* < 0.001, partial *η_p_*^2^ = 0.07. Separate univariate tests on the outcome variables revealed significant group differences in the amount of caring activities: *F*(1, 209) = 15.14, *p* < 0.001, *η_p_*^2^ = 0.07. The group difference in family functioning was not significant.

### 3.2. Variables Linked to Life Satisfaction in Young Carers

A hierarchical regression analysis was conducted to assess how this study independent variables were associated with life satisfaction in YCs (see Table 4). When all variables were included (Step 3), the model accounted for 53.4% of the variance in life satisfaction. In this model, academic functioning (β = 0.31, *p* < 0.01), satisfaction with social support (β = 0.36, *p* < 0.01), and the negative impact of caring (β = −0.38, *p* < 0.01) were significantly associated with life satisfaction.

### 3.3. Variables Linked to Negative Impact of Caring in Young Carers

The next regression analysis explored the extent to which this study independent variables were associated with the negative impact of caring in YCs (Table 5). When all variables were included (Step 3), the model accounted for 33.0% of the variance in life satisfaction. In this model, family functioning (β = 0.30, *p* < 0.05) and the amount of caring activities (β = 0.38, *p* < 0.01) were significantly associated with the negative impact of caring.

### 3.4. Variables Linked to Life Satisfaction in Non-Carers

For the final analysis, we tested the extent to which this study independent variables were associated with life satisfaction in the NCs group (Table 6). When all variables were included, the final model (Step 3) accounted for 46% of the variance in life satisfaction. In this model, academic functioning (β = 0.27, *p* < 0.05), social support (β = 0.28, *p* < 0.01), and family functioning (β = −0.31, *p* < 0.05) were significantly associated with life satisfaction.

## 4. Discussion

YCs have been the subject of study for several decades, with findings within the respective literature contributing significantly to the development of supportive policies and practices [26]. Similar findings on the prevalence and challenges faced by YCs in their role of caring for ill/disabled relatives and friends have been reported in various countries [17,39]. Available data indicate that out of every 100 adolescents, around 6 to 8 are YCs, and some of them require community and professional support [39]. This suggests that there may be 2–3 YCs in every classroom, regardless of country. However, to date, there has been no research into Portuguese YCs [77,78].

Demographic and socio-economic challenges in Portugal suggest that a substantial number of children and young people in the country may be YCs, even if they remain largely unidentified. These demographic changes include factors such as longer life expectancy and an aging population, a rising number of single-parent families, delayed parenthood, and a growing prevalence of chronic and mental health conditions. Notably, 95% of those affected by such conditions live in family settings [15], and cultural norms emphasize the central role of the family in the caregiving system. Portuguese health professionals have previously acknowledged the existence of YCs [77,78,79], although primarily focused on the island of Madeira, and no direct data has ever been collected concerning Portuguese YCs. As such, the present study can be seen as a pioneering effort, representing the first significant research initiative focused on Portuguese YCs. Its primary contribution lies in raising awareness of this group’s needs, as the mere recognition of the existence of Portuguese YCs is an important outcome itself.

Our study involved screening over 9000 Portuguese adolescents, with more than 900 students voluntarily disclosing their experiences of caring for their ill or disabled relatives. This represents groundbreaking preliminary data that underscores the importance of recognizing and identifying Portuguese YCs wherever they may be and advocating for research and policies to address their unique needs. The data presented in this study indicate a higher proportion of Portuguese YCs compared to some other countries that have established research and policy initiatives focused on this population [80,81].

This study represents an early step in acknowledging the reality of Portuguese YCs. Our results also offer valuable insights into the factors that contribute to better outcomes for YCs in terms of their wellbeing and life satisfaction, addressing specific developmental challenges. Our examination of YCs life satisfaction, which takes into consideration their social support, family dynamics, academic functioning, and their experiences and perceptions of caring activities, offers a significant contribution to understanding the impact of their experiences on overall wellbeing. As anticipated, our findings confirmed that YCs tend to have lower life satisfaction than NCs [82]. This result aligns with existing concerns about the wellbeing of YCs and the consequences of caring for their health and opportunities, which have been previously documented [22,30,31,32,33,34,82]. YCs often experience feelings of worry and hypervigilance related to their caring responsibilities [83,84], and being a carer is consistently associated with poorer mental health outcomes, including depression and anxiety [21]. Moreover, the presence of mental health issues is strongly associated with reduced satisfaction with life [85].

The experiences of caregiving have been shown to have significant impacts on the wellbeing of YCs. The range of personal challenges stemming from extensive and continuous caregiving can have adverse effects on YCs physical and mental health [3,4,12,18,19,22,46]. These impacts have also been observed to extend to social exclusion and difficulties in school [3,27,39], which, in turn, can impair development and wellbeing [26,39]. It is important to emphasize that our research findings indicate that YCs exhibit lower life satisfaction than their NCs peers. Furthermore, YCs academic functioning, their satisfaction with social support, and the negative subjective impact of caregiving were found to be linked to life satisfaction. This underscores the important role of social support in the wellbeing of YCs [3,40,42]. YCs frequently grapple with a lack of support and recognition, particularly when they are not aware of their caregiver identity and/or when those in their immediate environments are unaware of their caregiving responsibilities [27,84]. Our research suggests that this lack of recognition and support is linked to YCs feeling less satisfied with their lives, especially when their teachers and peers are unaware of or fail to understand their circumstances, which can lead to misinterpretations of behaviors and leave YCs more socially isolated and emotionally lonely [19]. Conversely, being acknowledged, identified, and understood by others as caregivers and receiving appropriate support from peers and school staff may significantly enhance YCs life satisfaction [19].

Schools and the support they offer play a key role in improving the life satisfaction of YCs and can be a protective factor. Our results suggest that, beyond their subjective experience of caring, YCs life satisfaction may be dependent on their ability to maintain a fulfilling role as students. Given that their caring responsibilities demand significant time and energy from YCs, it often leaves them with less time to devote to their student role, leading to a sense of role overload [17]. Conversely, when their caring role does not impose an excessive burden and they can effectively fulfill their responsibilities as students, including achieving desired grades, punctuality, regular attendance, and diligent home studying, their life satisfaction increases. This aligns with research indicating that YCs often experience negative emotional consequences (extreme outbursts of anger, sadness, and disappointment), especially when they struggle to meet educational requirements and deadlines [46]. Consequently, our results highlight that adolescents’ roles as students significantly contribute to their life satisfaction, regardless of whether they assume a caring role. Life satisfaction is a comparative process in which individuals evaluate their lives against self-imposed standards [86], encompassing dimensions such as family, friends, and school [87]. During adolescence, meeting basic psychological needs at school has been linked with wellbeing and life satisfaction [64,88,89], emphasizing the crucial role of the school context and student role in adolescents’ lives [90]. Thus, support from schools and teachers assumes a vital role in the wellbeing and development of YCs. It is noteworthy that many YCs find going to school a source of respite from caring, providing them the possibility to socialize, have fun, and learn [36].

While family functioning was not initially found to be associated with life satisfaction, when controlling for caring variables, family functioning was found to have a significant association with the negative impact of caregiving, which in turn also predicts YCs life satisfaction. Thus, the negative impact of caregiving on YCs can be partially explained by their family functioning and the extent of their caregiving activities. Family functioning has been relatively underexplored in the literature on YCs. However, Bou’s study [22] has indicated that when YCs feel supported and acknowledged by their family members, including the care recipient, they tend to report improvements in family closeness and their relationship with the care recipient. Conversely, when YCs lack such support, they often view their care negatively [22]. Furthermore, recognition of the caring role and support in caring, including from family members and the care recipient, have been associated with better wellbeing [27]. Thus, low family resources, ineffective communication within the family, and family difficulties carry predictive weight for the negative impact of caring. This result emphasizes the protective role of family functioning in the wellbeing of YCs. Adaptive family functioning has been linked to positive development among young people, particularly in terms of mental health, self-esteem, and life satisfaction. Conversely, issues in family functioning can significantly impact the adjustment of young people and have been associated with symptoms of depression and anxiety [67].

Non-carers exhibited considerable variability in the domestic and familial tasks they performed, as expected. For NCs, satisfaction with life was significantly associated with academic functioning, satisfaction with social support, and family functioning, but not the amount of activities they undertake. In short, caregiving responsibilities and their associated meaning have a negative impact on the life satisfaction of YCs but not on NCs, as one might expect [35,57]. A YCs caregiving duties may encompass a wide range of tasks, including domestic tasks, household management, financial management, personal care, emotional support, and sibling assistance [41]. The cumulative nature of tasks has a significant impact on the lives of YCs [39,91] especially in cases like our sample, where the more tasks a YC is responsible for, the greater the negative impact of caring.

Irrespective of the amount of care, a lower quality of relationship with the person being cared for, as well as the emotional and physical demands of providing care (surely intertwined with the multifaceted and varied impact of family illness, demanding additional studies), are factors associated with increased stress and consequently, a greater impact of care on life satisfaction. Therefore, this result emphasizes the importance of considering these factors when designing programs aimed at enhancing the life satisfaction of YCs. Moreover, the significance of the subjective meaning of caring activities as opposed to the mere quantity of caring tasks highlights the need for a more reflective and psychologically informed approach to understanding YCs experiences, which goes beyond the objective reporting of tasks.

In addition to the negative impacts of caring on the mental and physical health of YCs, it is important to recognize that the caring role may have positive consequences [24,25,40], such as fostering maturity and competence [39]. Therefore, programs aimed at enhancing the life satisfaction of YCs should also address the positive consequences of caregiving [3,19,41,42,43], especially by helping them to develop self-compassion and empathy [3,24,44,45], feelings of fulfillment [22] or accomplishment [49], and a sense of purpose [38]—all of which contribute to positive adaptations and positive subjective feelings related to caring. Not only can caring have positive outcomes, but these can be further enhanced through the principles of positive psychology. Furthermore, promoting and increasing life satisfaction, self-compassion, fulfillment, and flourishing can play a significant role in mitigating the negative impact of caregiving [22], which has been shown to influence YCs life satisfaction. As Gough and Gulliford [49] suggest, adopting a benefit-seeking orientation, where positive growth is derived from adversity, can make YCs more resilient and help them find a sense of satisfaction and fulfillment in their roles. This approach can equip them with practical coping strategies and a strong sense of agency and connection, which, in turn, promote better outcomes for YCs. Benefit discovery has been linked to improved mental well-being both directly and indirectly through better coping and a reduced sense of helplessness [50]. Recognizing and supporting YCs involves more than just identifying and addressing their vulnerabilities to bring them on par with NCs. In fact, it is an opportunity to view the caring role as a positive avenue for personal growth and development, extending across the life span and within various relational contexts. This encompasses family development stages as well as the cultivation of positive, mutually rewarding, and healthy caregiving relationships.

## 5. Conclusions

The present study represents one of the first studies on YCs in Portugal. Studying hidden groups, particularly when they involve children, presents methodological complexities. It involves addressing intricate ethical considerations and informed parental consent requirements. In the Portuguese context, these challenges are amplified by the low awareness of YCs among the general public and professionals, including policymakers, teachers, and communities. Many YCs themselves do not recognize or identify as ‘carers’ but rather view themselves as children living with parents and other family members who may have some illness or condition, which requires them to “help at home”.

The identification and recruitment of YCs present notable challenges, and there is not a universally agreed-upon approach to locating or quantifying them [92]. Internationally, YCs have been identified through national censuses, large surveys, longitudinal studies, and by children self-identifying as YCs. For a country like Portugal, which is still in the early stages of YCS research and policy development, the question arises: What should come first, awareness-raising and support activities for YCs, or research on/with YCs?

Following Hanson, et al. [93], we would suggest that awareness-raising efforts targeted at key groups, such as healthcare workers, teachers, social workers, etc., without substantial research evidence about the extent and nature of young caring are unlikely to be effective. Our strategy, both in this research and beyond, has been to systematically disseminate knowledge about YCs, facilitating awareness among the general public, academics, and policymakers. This approach serves to put Portuguese YCs “on the map” and helps establish a research agenda. The ‘Projecto Jovens Cuidadores’ has served as a multi-level pilot structure involving all these institutions, contributing to awareness-raising and the identification of YCs needs and characteristics. It also highlighted the importance of involving adolescents, their families, teachers, and peers in a community school-based approach when it comes to accessing and addressing their needs. Talking about their reality in every classroom and every school we could visit has proven to be a good way to highlight and empower these young people. It also contributes to a positive view of this caregiving role and calls on the entire community to support the YCs.

The research presented in this study sheds light on the experiences of YCs in Portugal, bringing them out of obscurity. It highlights the factors associated with YCs life satisfaction with life, as well as those that are associated with negative impacts and outcomes. This study serves as a foundational step toward the development of policies and practices in Portugal. Policy and interventions should be designed to accentuate the positive aspects and reduce or mitigate the negative factors. Additionally, valuable lessons can be drawn from international advances in policies and practices. Research into YCs in Portugal is in its infancy, just as the development of policy and practice is. There is undoubtedly a long road ahead, but this study marks a significant starting point.

## Figures and Tables

**Table 1:** Sociodemographic characteristics of the sample (*N* = 235).

	Young Carers		Non-Carers		Total Sample	
	(*n* = 106)		(*n* = 129)		(*n* = 235)	
Characteristic	*n*	%	*n*	%	*N*	%
Gender						
Male	35	33.02	54	41.86	89	37.87
Female	71	66.98	69	53.49	140	59.57
Missing	0	0.00	6	4.65	6	2.55
Grade						
Grade 7–9 (Basic Education)	53	50	92	71.32	145	61.71
Grade 10–12 (Secondary Education)	53	50	37	28.68	90	38.29
Mother’s Highest Education Level						
Basic Education (Grade 1–9)	49	46.23	39	32.30	88	37.45
Secondary Education (Grade 10–12)	23	21.70	31	24.03	54	22.98
Higher Education	25	23.58	38	29.46	63	26.81
Missing	9	8.49	21	16.28	30	12.77
Father’s Highest Education Level						
Basic Education (Grade 1–9)	56	52.83	42	32.56	98	41.70
Secondary Education (Grade 10–12)	23	21.70	33	25.58	56	23.83
Higher Education	14	13.21	28	21.71	42	17.87
Missing	13	12.26	26	2.16	39	16.60
Family Structure						
Single-parent family	18	16.98	12	9.30	30	12.77
Two-parents family	33	31.13	86	66.67	119	56.40
Stepfamily	15	14.15	7	5.43	22	9.36
Extended family	36	33.96	15	11.63	51	21.70
Missing	4	3.77	9	6.98	13	5.53

**Table 2:** Young Carers caring characteristics (N = 106).

	*n*	%
Cared person		
Parent or similar (mother, father, stepmother, or stepfather)	44	41.5
Grandparent or great-grandparent	38	35.8
Sibling	16	15.1
Other relative	8	7.5
Number of cared persons		
One person being cared	91	85.8
More than one person being cared	15	14.1
Years of caring role: M(SD) = 4.55 (3.468), 0–16 years		
Up to 2 years	35	33.0
3 to 6 years	43	40.6
7 years or more	28	26.4
Amount of caring tasks (MACA-YC18)		
Low (00–09 points)	10	9.4
Moderate (10–13 points)	24	22.6
High (14–17 points)	40	37.7
Very high (18–36 points)	32	30.2
Hours on caring tasks per week		
Less than 7 h/week	26	24.5
7 a 14 h/week	30	28.3
Over than 14 h/week	16	15.1
Missing	34	32.1

**Table 3:** Mean (M) and standard deviation (SD) results regarding young carers and non-carers.

	Young Carers (*n* = 106)		Non-Carers (*n* = 129)		Between Group Differences	
	*M*	*SD*	*M*	*SD*	*F*	*p*
Satisfaction with life	4.66	0.88	4.93	0.66	5.10	0.045
Amount of caring activities	15.95	5.59	12.05	6.75	15.14	<0.001
Social support	3.41	0.72	3.64	0.69	3.55	0.061
Family functioning	2.26	0.82	2.02	0.77	2.17	0.142
Academic functioning	3.85	0.61	3.83	0.61	0.04	0.841
Negative impact of caring	3.40	3.48				

**Table 4:** Hierarchical regression for family functioning, academic functioning, satisfaction with social support, negative impact of caring, and amount of caring activities as predictors of life satisfaction in adolescent young carers (YCs), controlling for age (N = 57).

	β	95% CI		*t*	R^2^	ΔR^2^
		LL	UL			
Step 1					0.002	0.002
Age	−0.04	−0.31	0.23	−0.31		
Step 2					0.34	0.34 ***
Age	0.04	−0.19	0.27	0.33		
Family functioning	−0.34 **	−0.57	−0.11	−2.97		
Academic functioning	0.26 *	0.09	0.54	2.26		
Social support	0.32 **	0.03	0.49	2.78		
Step 3					0.53	0.19 ***
Age	0.11	−0.09	0.31	1.11		
Family functioning	−0.15	−0.37	0.06	−1.41		
Academic functioning	0.31 **	0.16	0.56	3.06		
Social support	0.36 **	0.11	0.51	3.57		
Negative impact of caring	−0.38 **	−0.61	−0.14	−3.20		
Amount of caring activities	−0.18	−0.41	0.40	−1.66		

*Legend:* * *p* < 0.05; ** *p* < 0.01; *** *p* < 0.001; CI—Confidence Interval.

**Table 5:** Hierarchical regression for family functioning, academic functioning, satisfaction with social support, and amount of caring activities as predictors of the negative impact of caring in YCs, controlling for age (N = 57).

	β	95% CI		*t*	R^2^	ΔR^2^
		LL	UL			
Step 1					0.03	0.03
Age	0.17	−0.09	0.44	1.27		
Step 2					0.21	0.18 *
Age	0.14	−0.11	0.39	1.10		
Family functioning	0.39 **	0.13	0.63	3.07		
Academic functioning	0.17	−0.08	0.42	1.36		
Social support	0.14	−0.11	0.39	1.12		
Step 3					0.33	0.13 **
Age	0.10	−0.140	0.33	0.82		
Family functioning	0.30 *	0.06	0.53	2.51		
Academic functioning	0.20	−0.04	0.43	1.70		
Social support	0.16	−0.07	0.40	1.41		
Amount of caring activities	0.38 **	0.13	0.62	3.12		

*Legend:* * *p* < 0.05; ** *p* < 0.01; CI—Confidence Interval.

**Table 6:** Hierarchical regression for family functioning, academic functioning, satisfaction with social support, and amount of caring activities as predictors of life satisfaction in adolescent non-carers (NCs), controlling for age (N = 82).

	β	95% CI		*t*	R^2^	ΔR^2^
		LL	UL			
Step 1					0.00	0.00
Age	−0.01	−0.23	0.21	−0.05		
Step 2					0.45	0.45 ***
Age	0.07	−0.48	−0.04	0.75		
Family functioning	−0.26 *	−0.70	−0.06	−2.36		
Academic functioning	0.30 **	0.09	0.50	2.85		
Social support	0.29 **	0.10	0.48	2.85		
Step 3					0.46	0.01 ***
Age	0.06	−0.12	0.23	0.63		
Family functioning	−0.31 *	−0.56	−0.06	−2.47		
Academic functioning	0.27 *	0.05	0.49	2.46		
Social support	0.28 **	0.09	0.48	2.91		
Amount of caring activities	0.08	−0.12	0.28	0.81		

*Legend:* * *p* < 0.05; ** *p* < 0.01; *** *p* < 0.001; CI—Confidence Interval.

## Data Availability

Data are unavailable due to privacy and ethical restrictions.
